# Membrane Topology and Heme Binding of the Histidine Kinases HrrS and ChrS in *Corynebacterium glutamicum*

**DOI:** 10.3389/fmicb.2018.00183

**Published:** 2018-02-09

**Authors:** Marc Keppel, Eva Davoudi, Cornelia Gätgens, Julia Frunzke

**Affiliations:** Institute of Bio- and Geosciences, IBG-1: Biotechnology, Forschungszentrum Jülich, Jülich, Germany

**Keywords:** *Corynebacterium glutamicum*, two-component systems, heme, heme-binding protein, transient heme sensing, ChrSA, HrrSA

## Abstract

The HrrSA and the ChrSA two-component systems play a central role in the coordination of heme homeostasis in the Gram-positive soil bacterium *Corynebacterium glutamicum* and the prominent pathogen *Corynebacterium diphtheriae*, both members of the *Corynebacteriaceae*. In this study, we have performed a comparative analysis of the membrane topology and heme-binding characteristics of the histidine kinases HrrS and ChrS of *C. glutamicum*. While the cytoplasmic catalytic domains are highly conserved between HrrS and ChrS, the N-terminal sensing parts share only minor sequence similarity. PhoA and LacZ fusions of the N-terminal sensor domains of HrrS and ChrS revealed that both proteins are embedded into the cytoplasmic membrane via six α-helices. Although the overall membrane topology appeared to be conserved, target gene profiling indicated a higher sensitivity of the ChrS system to low heme levels (< 1 μM). *In vitro*, solubilized and purified full-length proteins bound heme in a 1:1 stoichiometry per monomer. Alanine-scanning of conserved amino acid residues in the N-terminal sensor domain revealed three aromatic residues (Y^112^, F^115^, and F^118^), which apparently contribute to heme binding of HrrS. Exchange of either one or all three residues resulted in an almost abolished heme binding of HrrS *in vitro*. In contrast, ChrS mutants only displayed a red shift of the soret band from 406 to 418 nm suggesting an altered set of ligands in the triple mutant. In line with target gene profiling, these *in vitro* studies suggest distinct differences in the heme-protein interface of HrrS and ChrS. Since the membrane topology mapping displayed no extensive loop regions and alanine-scanning revealed potential heme-binding residues in α-helix number four, we propose an intramembrane sensing mechanism for both proteins. Overall, we present a first comparative analysis of the ChrS and HrrS kinases functioning as transient heme sensors in the *Corynebacteriaceae*.

## Introduction

Heme represents a ubiquitous cofactor of proteins and plays a vital role in a variety of cellular processes, including electron transport, oxygen transport, or oxidative stress responses (Poulos, 2007). Ferriprotoporphyrin IX (heme) is also the predominant form of iron in vertebrates and is consequently also exploited as an important iron source for – not only – pathogenic bacteria (Andrews, 2008; Cornelis et al., 2011). Elevated heme levels, however, cause toxicity by so far not satisfactorily settled mechanisms (Anzaldi and Skaar, 2010). Thus, the intracellular heme pool is typically tightly balanced by a complex and integrative regulatory network.

In bacteria, two-component systems (TCS) represent a ubiquitous principle used by the cells to sense and respond to a variety of different stimuli (Mascher et al., 2006). A prototypical TCS consists of a membrane bound histidine kinase (HK) with a unique sensing domain and a conserved, catalytically active kinase core and a cytoplasmic response regulator (RR) which can be phosphorylated and activated by the HK (Zschiedrich et al., 2016). In response to the stimulus, the HK is autophosphorylated at a conserved histidine residue. The following phosphoryl transfer to the RR typically leads to the activation of this protein, which then drives cellular adaptation, for example, by controlling target gene expression (Stock et al., 2000; Laub and Goulian, 2007).

In the Gram-positive soil bacterium *Corynebacterium glutamicum*, two paralogous TCS, HrrSA and ChrSA, have been reported to play a central role in the control of heme homeostasis (Frunzke et al., 2011; Bott and Brocker, 2012; Heyer et al., 2012). While the HrrSA system is crucial for utilization of heme as an alternative iron source by activating the expression of the heme oxygenase (*hmuO*) under iron limiting conditions (Frunzke et al., 2011), the TCS ChrSA is required to cope with elevated heme levels by activating the expression of a putative heme exporter *hrtBA* (Heyer et al., 2012). Significant cross-phosphorylation between these two systems has been demonstrated in previous studies (Hentschel et al., 2014) reflecting the striking similarity of the HisKA_3 domains of the sensor kinases HrrS and ChrS [pfam07730, ∼40% sequence identity (Finn et al., 2010)]. In contrast, the N-terminal, membrane anchored signal perception domains of the sensor kinases share a low sequence identity of only 8.5% (Supplementary Figure S1). Nevertheless, previous studies provided evidence that heme is perceived as a stimulus by both kinases (Frunzke et al., 2011; Heyer et al., 2012).

In nature, the predominant types of heme are heme *b* and heme *c*. While heme c is mostly found covalently bound to proteins, heme *b* is often non-covalently bound (Bowman and Bren, 2008; Brewitz et al., 2017). According to a bioinformatical analysis of 125 different heme-binding proteins, the top five residues with high frequencies in close proximity to the binding pocket are cysteine, histidine, phenylalanine, methionine, and tyrosine (Li et al., 2011). Overall, 31 different structural folds were reported for the protein–heme interaction, hinting to a very diverse mode of interaction between proteins and heme as ligand (Li et al., 2011).

In the ChrS homolog of *Corynebacterium diphtheriae*, a single tyrosine residue (Y61) was speculated to be involved in heme binding (Ito et al., 2009; Bibb and Schmitt, 2010). A further example of a heme-responsive TCS is the heme sensor system (HssRS), which has been studied in *Staphylococcus aureus* and *Bacillus anthracis* (Stauff and Skaar, 2009a,b). In this case, several amino acids were speculated to be involved in heme binding. However, the periplasmic loop domain of HssS represents a significant difference to HrrS and ChrS in *Corynebacteria.* In contrast to ChrS, heme binding and membrane topology of HrrS has not been studied so far.

Here, we performed a comparative sequence and biochemical analysis of the transmembrane domain of *C. glutamicum* HrrS and ChrS. Our data suggested that regardless of the low sequence identity of the sensor domain, both kinases are embedded into the cytoplasmic membrane via six α-helices. *In vitro* analysis of purified full-length proteins revealed that HrrS and ChrS bind heme in a 1:1 stoichiometry per monomer. Furthermore, three conserved aromatic residues (Y^112^, F^115^, and F^118^) were identified, which are of crucial importance for heme binding of HrrS. Conclusively, we provide insights into the organization of the sensor domain of these two closely related HKs and present a model for an intramembrane heme interface for signal perception.

## Materials and Methods

### Bacterial Strains and Growth Conditions

*Corynebacterium glutamicum* ATCC 13032 was used as wild type strain (Kalinowski et al., 2003) (Supplementary Table S1). For reporter studies, a BHI pre-culture (brain heart infusion, Difco BHI, BD, Heidelberg, Germany) was inoculated with cells from a fresh agar plate and incubated for 8–10 h at 30°C. Subsequently, cells were transferred into a CGXII (Keilhauer et al., 1993) preculture containing 2% (w/v) glucose and 0 μM FeSO_4_. These conditions have been optimized to achieve iron starvation. Protocatechuic acid (PCA) was present in the pre-culture, allowing the uptake of trace amounts of iron. After overnight growth, a CGXII main culture (containing hemin as iron source) was inoculated to an OD_600_ of 1. If necessary, 25 μg/ml kanamycin or 10 μg/ml chloramphenicol was added.

*Escherichia coli* was cultivated in Lysogeny Broth (LB) medium at 37 or 20°C for protein production. If necessary, 50 μg/ml kanamycin or 34 μg/ml chloramphenicol was added.

### Cloning Techniques and Recombinant DNA Work

Routine molecular biology methods were performed according to standard protocols (Sambrook and Russell, 2001). Chromosomal DNA of *C. glutamicum* ATCC 13032 was used as template for PCR amplification of DNA fragments was and prepared as described earlier (Eikmanns et al., 1994). DNA sequencing and synthesis of oligonucleotides was performed by Eurofins Genomics (Ebersberg, Germany).

For the construction of expression plasmids (Supplementary Table S1), DNA fragments were amplified with oligonucleotides given in Supplementary Table S2 and assembled into a vector backbone either by Gibson et al. (2009) or via standard restriction digestion and ligation. For the *phoA*/*lacZ* assays, pT7-5-*phoA* and pT7-5-*lacZ* were used as vector backbones. Both plasmids were kindly donated by the laboratories of G. Unden (Bauer et al., 2011).

Wild type and mutant versions of *hrrS* and *chrS* were expressed from the pkW3 plasmid backbone. The mutations were introduced via “QuikChange Lightning” site directed mutagenesis according to the supplier’s manual (Agilent Technologies, Santa Clara, United States). The origin plasmid was digested with *Dpn*I (digestion of methylated DNA) and the mutant plasmid was transferred into *E. coli* TOP-10. The introduced mutations were confirmed by sequencing.

### Alkaline Phosphatase and β-Galactosidase Assays

For both, the alkaline phosphatase (PhoA) and the β-galactosidase (LacZ) assay, *E. coli* TG-1 was transformed with truncated *hrrS* or *chrS* versions fused to either *phoA* or *lacZ* in the pT7-5 vector and grown overnight in LB medium containing kanamycin (50 μg/ml). The overnight culture was diluted 1:50 in fresh medium and grown for 1.5 h (∼OD_600_ = 0.1). Subsequently, expression of the *phoA*/*lacZ* fusions was induced with 1 mM IPTG for 1 h. The activities of PhoA and LacZ fusions were measured as described as follows: for the PhoA assay, 100 μl of the *E. coli* cells were harvested and resuspended in 300 μl of 1 mM Tris–HCl (pH = 8.0). Cells were permeabilized by the addition of 25 μl 0.1 % (w/v) SDS and 25 μl chloroform to allow both substrates (*p*-nitrophenyl phosphate and 2-nitrophenyl β-D-galactopyranoside) to enter the cells. Samples were incubated for 20 min at room temperature (Manoil, 1991). Subsequently, 200 μl of the upper phase was transferred to a microtiter plate and the reaction was started by the addition of 25 μl of a 5 mg/ml *p*-nitrophenyl phosphate solution (in 1 mM Tris–HCl, 5 mM MgCl_2_, pH 8.0). For background measurement and normalization, Abs_600_ and Abs_500_ were measured immediately. The plates were incubated at 28°C for up to 2 h and the yellow color change was determined by measuring the Abs_420_.

For LacZ, the protocol was derived from a protocol published by Miller (1992): 100 μl of the *E. coli* culture was harvested and resuspended in 300 μl buffer Z (60 mM Na2HPO4⋅12H2O, 40 mM NaH2PO4⋅H2O, 1 mM MgSO4⋅7H2O, 10 mM KCl, and 100 mM freshly added DTT).

The cells were permeabilized and transferred to a microtiter plate. The reactions were started by addition of 25 μl of a 5 mg/ml 2-nitrophenyl β-D-galactopyranoside solution (in 100 mM K_2_HPO_4_, pH = 7.0). Measurements were performed as described for PhoA above. Miller units for both enzymes were calculated according to Supplementary Formula S1.

### Reporter Assays

For fluorescent reporter assays a 20 ml pre-culture of CGXII minimal medium containing 2% (w/v) glucose was inoculated from a 5 ml BHI culture of *C. glutamicum* carrying the reporter plasmid (e.g., pJC1_P*_hmuO_-eyfp* or pJC1_P*_hrtBA_-eyfp*) (Hentschel et al., 2014) and expressing a *hrrS* or *chrS* mutant gene (fused to the *flag*-gene) from the plasmid pKW3 under control of the native promotor. To cause iron starvation, no additional iron source was added to the CGXII medium (PCA was present in the pre-culture, allowing the uptake of trace amounts of iron). The cells were incubated overnight at 30°C in a rotary shaker and grew to an OD_600_ of ∼10–20. Reporter assays in microtitre scale were performed in the BioLector system (m2p-labs GmbH, Aachen, Germany). Therefore, 750 μl CGXII medium containing 2% (w/v) glucose and 2.5 μM hemin were inoculated from the second pre-culture with iron-starved cells to an OD_600_ of 1 and cultivated in 48-well Flowerplates^®^ (m2p-labs GmbH, Aachen, Germany) at 30°C, 95% humidity, 1200 r.p.m. For the hemin stock solution, hemin (Sigma–Aldrich, Munich, Germany) was dissolved in 20 mM NaOH to a concentration of 2.5 mM and diluted in water to 250 μM. Biomass production of the growing cells was determined as the backscattered light intensity of sent light with a wavelength of 620 nm (signal gain factor of 12). For the measurement of eYFP fluorescence, the chromophore was excited at 510 nm and emission was measured at 532 nm (signal gain factor of 50). The specific fluorescence corresponds to the total eYFP fluorescence signal in relation to the backscatter signal and is given in arbitraty units (a.u.) (Kensy et al., 2009).

### Overproduction and Purification of Full-Length Histidine Kinases

For the overproduction of HrrS-CStrep and ChrS-CStrep, *E. coli* BL21(DE3) was either transformed with the vectors pET24b-*hrrS-Cstrep*, pET24b-*chrS-Cstrep* or a mutated version and cultivated in 500 ml LB medium at 37°C and 100 rpm. At an OD_600_ of ∼0.7, expression was induced by the addition of 0.5 mM IPTG. After 12–16 h at 20°C, cells were harvested by centrifugation (4000 × *g* at 4°C, 10 min). The cell pellet was stored at -20°C. For protein purification, the cell pellet was resuspended in buffer W (100 mM Tris–HCl, pH = 8.0, 250 mM NaCl), containing “complete” protease inhibitor cocktail (Roche, Germany). Cells were disrupted by passing a French pressure cell (SLM Ainco, Spectronic Instruments, Rochester, NY, United States) three times at 207 MPa. The cell debris was removed by centrifugation (6900 × *g*, 4°C, 20 min), followed by an ultracentrifugation of the cell-free extract for 1 h (150,000 × *g*, 4°C). Pellets containing the membrane fraction were resuspended in 3 ml buffer W containing protease inhibitor. For solubilization of HrrS-CStrep and ChrS-CStrep, the membrane fraction was incubated with 1% n-Dodecyl-β-D-Maltoside (DDM) (Biomol, Germany) at 25°C on a rotary shaker for 1 h. After a second ultracentrifugation step (0.5 h, 150,000 × *g*, 4°C), solubilized HrrS-CStrep and ChrS-CStrep present in the supernatant were purified by strep-tactin affinity chromatography (1 ml column volume, CV). Equilibration was performed with 15 CV buffer W containing 0.03% DDM. After washing with 15 CV of buffer W containing 0.03% DDM, CStrep-proteins were eluted with 5 CV of buffer W containing 0.03% DDM and 15 mM desthiobiotin. Fractions containing the desired Strep-tagged proteins were pooled, and the buffer was exchanged against HEPES-buffer (20 mM HEPES, pH 7.5, 20 mM KCl, 20 mM MgCl2, 200 mM NaCl) using a PD10 desalting column (GE Healthcare, Munich, Germany). The purified proteins were kept at 4°C and immediately used for heme-binding studies. Purity of protein samples was analyzed on a 12% SDS-polyacrylamide gel, which was afterward stained with Coomassie brilliant blue (G250, VWR Chemicals, Pennsylvania, United States). The protein concentration was determined using the extinction coefficients [𝜀(HrrS) = 35535, 𝜀(ChrS) = 51575] with a nano-spectrophotometer at 280 nm. In our elution fractions, the DDM interfered with other assays such as Bradford protein assay or Pierce BCA Protein Assay (Thermo Fisher, Waltham, United States).

### Western Blot Analysis

The production of HrrS and ChrS flag-tag variants was verified by western blot analysis. *C. glutamicum* Δ*hrrS* Δ*chrS* strains expressing a *hrrS* or *chrS* mutant gene (fused to the *flag*-gene) from the plasmid pKW3 under control of the native promotor were cultivated as described in the Section “Bacterial strains and growth conditions.” After 12 h, the cells were harvested and disrupted by passing a French pressure cell. Subsequently, 20 μg of protein extract were resuspended in SDS sample buffer (124 mM Tris–HCl, pH = 6.8, 20% (v/v) glycerol, 4.6% (w/v) SDS, 1.4 M β-mercaptoethanol, 0.01% (w/v) bromophenol blue) and the proteins separated by SDS-gel electrophoresis (12% separating gel, Bio-Rad Laboratories, Inc., United States). The gel was electroblotted via a Transblot Semi-Dry Transfer Cell (Bio-Rad Laboratories, Inc., United States) to a nitrocellulose membrane (Amersham^®^ Hybond-ECL Membranes, GE Healthcare, Germany) in Towbin-Blotpuffer (25 mM Tris, 192 mM Glycin, 20 % (v/v) Methanol). Transfer was carried out for 45 min (15 V). The membrane was blocked for 1 h in TBST (20 mM Tris–HCl pH = 7.5, 500 mM NaCl, 0.05 % (v/v) Tween 20) with 5 % (w/v) skim milk powder and subsequently washed in TBST and for the specific detection of FLAG-tagged proteins. Membranes were incubated for 1 h in ANTI-FLAG^®^-antibody (produced in mouse, 5 μg/ml, Sigma–Aldrich, Munich, Germany). After washing in TBST (3×) the membrane was incubated in a 1:10000 dilution of peroxidase-coupled anti-mouse secondary antibody for 1 h (GE Healthcare, Germany). For the detection of peroxidase activity, the “Amersham ECL-Select Western Blotting Detection” regent was used (GE Healthcare, Germany) and analyzed with the LAS-3000 mini (Fuji Photo Film Co., Tokyo, Japan).

### Heme-Binding Assays with Purified Protein

To assess the heme binding of purified kinases, we proceeded as following: after cell disruption and removal of cell debris by centrifugation, the cleared lysate of *E. coli*, overproducing HrrS or ChrS, was incubated with 50 μM hemin for 1 h at 4°C prior to ultracentrifugation of the cell-free extract for 1 h (150,000 × *g*, 4°C). Subsequently, the proteins were purified as described above. The purified protein was then analyzed via UV-visual spectroscopy: The absorption from 280 to 600 nm was determined using the UV/Visible double beam spectrophotometer UV-1601 PC (Shimadzu, Kyoto, Japan). For *in vitro* binding assays, purified protein (no pre-incubation with hemin) was added to buffer containing hemin concentrations from 0.5 to 36 μM and incubated for 5 min at room temperature. Subsequently, the absorption from 280 to 600 nm was determined. The samples were then referenced to respective hemin concentrations in buffer without protein.

### *In Vitro* Phosphorylation Assays

The autophosphorylation of both full-length HKs was determined as follows: immediately after purification, both kinases were incubated with 0.25 μM[γ^-33^P]-ATP (10 mCi/ml; PerkinElmer, United States) mixed with 80 μM non-radioactive ATP. The mixture was incubated for up to 30 min and at different time points 7 μl aliquots were removed, mixed with an equal volume of 2 × SDS loading buffer (124 mM Tris–HCl, pH = 6.8, 20 % (v/v) glycerol, 4.6 % (w/v) SDS, 1.4 M β-mercaptoethanol, 0.01 % (w/v) bromophenol blue) and kept on ice. Without prior heating, the samples were subjected to an SDS–PAGE (12% separating gel, Bio-Rad Laboratories, Inc., United States). Dried, gels were exposed on storage phosphor imaging films (Fuji Photo Film Co., Tokyo, Japan) and analyzed with a Typhoon Trio Scanner (GE Healthcare, Germany) after 24 h.

## Results

### Transmembrane Organization of HrrS and ChrS Is Highly Conserved

The HrrSA and ChrSA systems represent two homologous TCS involved in heme-responsive gene regulation in *C. glutamicum*. Whereas the cytoplasmic domains of the HrrS and ChrS kinases show a high sequence identity (39.4% in the HisKA_3 domains), the N-terminal sensor domains share only minor conservation at the sequence level (8.5%, Supplementary Figure S1). In order to unravel mechanistic differences or similarities in signal perception, we analyzed the transmembrane topology of both N-terminal sensor domains. For this purpose, we used online prediction tools in combination with an experimental approach based on PhoA/LacZ fusions. For the prediction of membrane spanning α-helices different analysis tools were applied, including TopPredII (Claros and von Heijne, 1994), TMPred (Hofman and Stoffel, 1993), Hmmtop (Tusnady and Simon, 2001), Minnou polyview (Porollo et al., 2004), CBS TMHMM (Krogh et al., 2001), DAS (Cserzo et al., 1997) Mpex (Snider et al., 2009), TOPCONS (Tsirigos et al., 2015), and Phobius (Kall et al., 2007).

As indicated in Supplementary Table S3, five out of nine programs suggested five transmembrane helices (TMHs) for ChrS, whereas three tools predicted six TMHs. The outcome of this analysis was even more diverse in the case of HrrS, where predictions ranged from 3 to 7 TMHs for the HrrS sensor domain – highlighting the importance of experimental verification.

**FIGURE 1:**
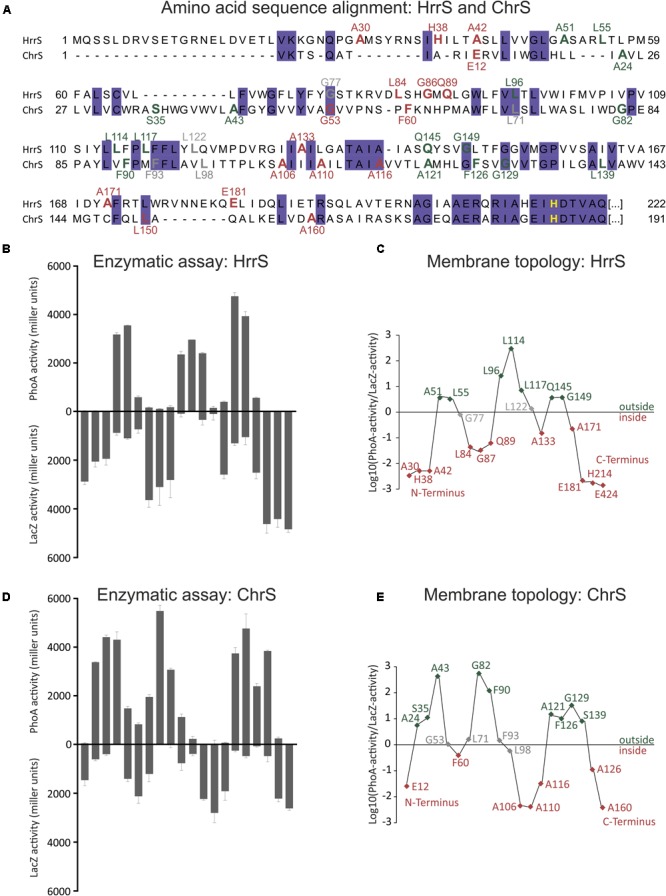
Experimental analysis of HrrS and ChrS membrane topology. **(A)** Sequence alignment of the N-terminal sensor domain of *C. glutamicum* HrrS and ChrS. A total of 20 truncated versions of HrrS or ChrS fused to the alkaline phosphatase (PhoA) or the β-galactosidase (LacZ) at the indicated amino acids positions were produced in *E. coli* TG-1 **(A)**. Based on the results indicated in **(B–E)** fusion residues are marked in red (inside) or green (outside). **(B)**+**(D)**: Enzymatic activities of 20 PhoA (up) and LacZ (down) fusions of HrrS and ChrS (given in miller units). The enzymatic activity of all fusion proteins was measured as described in the Section “Materials and Methods.” Data represent average values of three independent biological replicates. **(C)**+**(E)**: Log_10_ of the PhoA-activity/LacZ-activity ratios. The graph outlines the membrane topology of the histidine kinases (HKs) HrrS and ChrS: a ratio of > ∼0.5 indicates an extracellular and < ∼-0.5 a cytoplasmic localization.

For each kinase gene, *hrrS* and *chrS*, several truncated versions were fused to the *phoA* and *lacZ* genes at their 3′-end, encoding the alkaline phosphatase (PhoA) or the β-galactosidase (LacZ), respectively (**Figure 1A**). By expressing these fusion genes in *E. coli* TG-1, C-terminally truncated forms of HrrS and ChrS were obtained, that are linked to intact PhoA or LacZ enzymes. While PhoA displays high enzymatic activity if located in the periplasm, LacZ is only active if the enzymatic part of the protein is accessible in the cytosol (Manoil, 1991). Positions of the fusion constructs were distributed over the N-terminal sensor domain while focusing on residues close to predicted TMHs. Remarkably, this analysis revealed that both kinases, HrrS (**Figures 1B,D**) and ChrS (**Figures 1C,E**), are embedded into the cytoplasmic membrane via six TMHs. Thus, despite their minor sequence similarity, HrrS and ChrS share a conserved membrane topology. Referring to the results of the topology prediction tools, only TOPCONS (Tsirigos et al., 2015) and HMMTOP (Tusnady and Simon, 2001) were in line with our experimental data for both HrrS and ChrS.

### Target Gene Profiling Revealed a Higher Sensitivity of ChrS toward Heme in Comparison to HrrS

**FIGURE 2:**
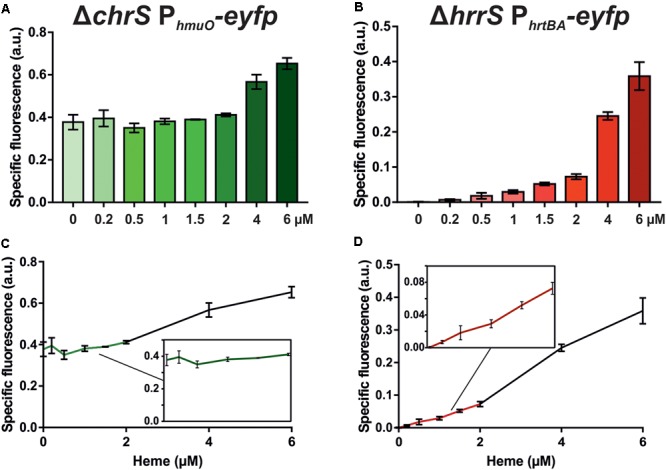
HrrS and ChrS target promoter profiling in response to heme. The *C. glutamicum* mutant strains Δ*chrS* or Δ*hrrS* were transformed with the target gene reporter pJC1_P*_hmuO_-eyfp* or pJC1_P*_hrtBA_-eyfp*, respectively. Cells were cultivated in a microbioreactor system (Biolector) in CGXII minimal medium with 2% (w/v) glucose containing 0–6 μM hemin. The maxima of the specific fluorescence after 7 h (P*_hmuO_*, **A** and **C**) or 3 h (P*_hrtBA_*, **B** and **D**) are shown. The specific fluorescence of mutant strains displaying background reporter levels was subtracted from the measurement (Δ*hrrSA* for P*_hmuO_-eyfp* and Δ*chrSA* for P*_hrtBA_-eyfp*). The reporter output over the time course of the experiment is shown in Supplementary Figure S2.

As a next step in the comparative analysis of HrrS and ChrS, we performed a profiling of target gene activation in response to heme as a stimulus. To avoid cross-phosphorylation between the systems and to focus on pathway activation mediated by only one of the two HKs, the deletion strains Δ*chrS* and Δ*hrrS* were transformed with the respective target gene reporter constructs (pJC1_P*_hmuO_-eyfp* and pJC1_P*_hrtBA_-eyfp*, respectively) (Hentschel et al., 2014). In the case of HrrS, the respective target promoter P*_hmuO_* already revealed a high background output caused by the iron limiting conditions leading to DtxR derepression (Wennerhold and Bott, 2006) (**Figure 2**, full spectra in Supplementary Figure S2). Heme-dependent activation via HrrS was only observed for heme concentrations above 4 μM resulting in a 1.5- to 1.7-fold induction (± 0.08/± 0.13) of the reporter (**Figure 2A**). In contrast to that the ChrS target gene reporter P*_hrtBA_-eyfp* (heme detoxification) showed almost no background activity and displayed a sensitive induction profile already at low (< 1.5 uM) heme concentrations, with a fold change of about 8 in the lower micromolar range (± 2.2, 0.2–1.5 μM) (**Figure 2B**). These findings are in agreement with previous data (Hentschel et al., 2014), suggesting a very sensitive response of the ChrSA system toward low heme levels, in contrast to the HrrSA system which facilitates heme utilization as an iron source, but only under iron limiting conditions (**Figures 2C,D**). Taken together, these results confirmed the heme-responsive activation of the HKs, but also provided evidence for differences in signal perception and ligand-binding affinity.

### HrrS and ChrS Are Heme-Binding Proteins

**FIGURE 3:**
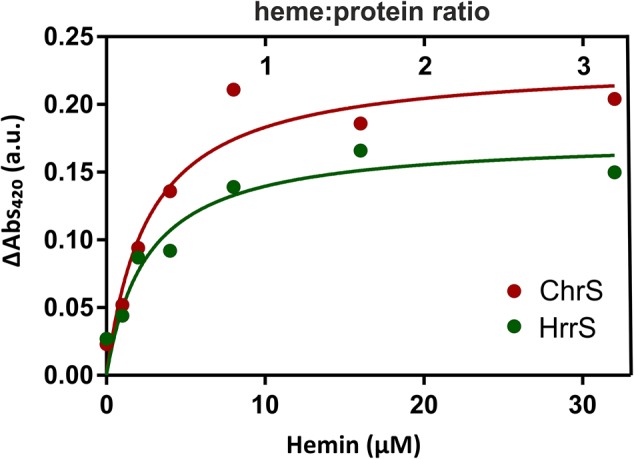
UV-Vis analysis of the heme-binding properties of HrrS and ChrS. For hemin-binding assays, different amounts of hemin were titrated to 10 μM purified HrrS or ChrS to a final concentration of 0, 2, 4, 8, 16, and 32 μM. The mixture was incubated for 5 min at RT and then analyzed by UV-visual spectroscopy. The resulting absorption was referenced against the absorption of buffer containing only DDM micelles but no protein. The graph shows the maxima of the soret peaks at 420 nm.

In the following, we performed *in vitro* studies to test whether both kinases bind heme via their N-terminal sensor domain. Therefore, both proteins were purified from *E. coli* BL21 (DE3) by the means of an N-terminal Streptactin tag (see the section “Materials and Methods”). Subsequently, 10 μM of solubilized and purified protein was mixed with different concentrations of hemin and analyzed by ultraviolet-visible spectroscopy (UV-Vis). Resulting spectra with the characteristic soret-peak at 406–420 nm were referenced against a buffer control, containing only the DDM micelles, in the absence of protein. The maximum values of the characteristic soret-peaks of heme/protein interaction at 420 nm are shown in **Figure 3**.

In our assay, both HrrS and ChrS showed heme-binding properties and the absorbance ΔAbs_420_ saturated slightly above 8 μM suggesting that both kinases bind heme in a 1:1 ratio (see full spectra in Supplementary Figure S3). These data, thus, emphasize that ChrS and HrrS contain a single heme-binding site per monomer.

### Identification of Putative Heme-Binding Residues by Alanine Scanning of HrrS

**FIGURE 4:**
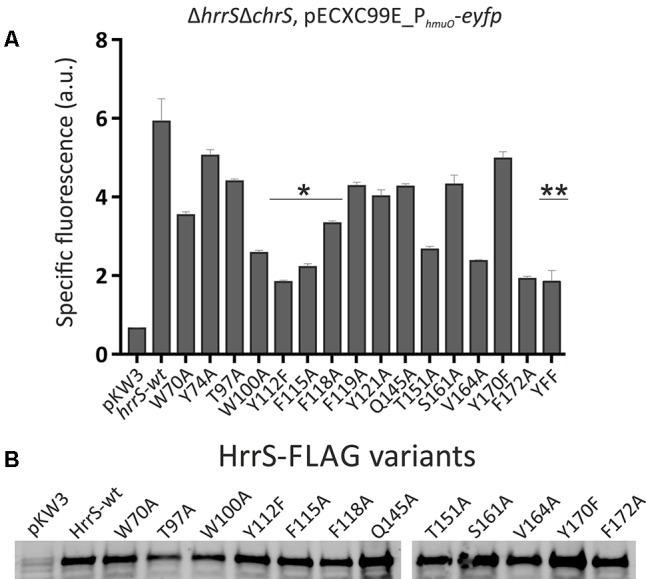
Alanine scanning of the N-terminal transmembrane domain of HrrS revealed putative heme-binding residues.**(A)** The *C. glutamicum* mutant strain Δ*hrrS*Δ*chrS* was transformed with the target gene reporter pECXC99E_P*_hmuO_-eyfp* and the pkW3 plasmid either containing wild type *hrrS* under its native promoter (wt) or 1 of 15 *hrrS* variants, encoding the HK with a single amino acid exchange. All proteins contained a C-terminal FLAG tag fusion for western blot analysis. Cells were cultivated in a microbioreactor system (Biolector) in CGXII minimal medium with 2% (w/v) glucose containing 2.5 μM hemin. The specific fluorescence after 8 h is shown. ^∗^Highlights three aromatic residues inside of helix 4 which were further investigated in following experiments. ^∗∗^Activation of the target gene reporter by the triple mutant HrrS^Y 112A-F115A-F118A^. **(B)** Western blot of HrrS variants (20 μg raw protein extract) using an anti-FLAG^®^ primary monoclonal ANTI-FLAG M2 antibody (5 μg/ml) and a peroxidase coupled anti-mouse secondary antibody.

Subsequently, we aimed at the identification of amino acid residues potentially involved in heme binding. Therefore, several conserved residues in the transmembrane domain of HrrS were exchanged against alanine to disrupt the heme-protein interface. The chosen amino acid residues are conserved among HrrS orthologs of several *Corynebacteriaceae* species (Supplementary Figure S4) and located within the N-terminal sensor domain of the kinase. Proper synthesis of most resulting proteins in *C. glutamicum* was confirmed by western blot analysis (**Figure 4B**). The heme-responsive activation of HrrS was monitored by using the P*_hmuO_*-*eyfp* target gene reporter described earlier (Hentschel et al., 2014).

Proteins were produced in a *C. glutamicum* Δ*hrrS*Δ*chrS* mutant strain to avoid cross-activation by the respective non-cognate kinase (Hentschel et al., 2014). The *C. glutamicum* Δ*hrrS*Δ*chrS* strain was transformed with the reporter plasmid pECXC99E_P*_hmuO_-eyfp* (chloramphenicol resistance) and complemented with pKW3 (kanamycin resistance) carrying either wild type *hrrS* under the control of its own promoter (P*_hrrS_-hrrS*) or one of the 15 *hrrS* single residue exchange variants. Whereas the empty vector control only displayed background levels of fluorescence, a significant reporter output could be restored by complementation with wild type *hrrS* (**Figure 4A**, full spectra shown in Supplementary Figure S5).

With this approach several residues were identified, that, upon exchange against alanine, led to a significantly reduced reporter output [up to 66 % reduction (Y112) compared to wild type HrrS]. Particularly interesting is a cluster of four aromatic amino acids inside or in close proximity of α-helix 4: W^100^-Y^112^-F^115^-F^118^. Indeed, previous studies revealed that tyrosine and phenylalanine residues are prime candidates for heme-interaction (Li et al., 2011). However, the tryptophan W^100^ appeared to be of structural importance for HrrS. This residue is located in the middle of α-helix three and purification of this variant led to a highly unstable and aggregated eluate, while HrrS-wt or other variants were purified as stable protein. Furthermore, HrrSW100A and HrrST97A show slightly reduced protein amounts in our western blot analysis (**Figure 4B**). Consequently, we concentrated on the triple residue, intermembrane motive Y^112^-F^115^-F^118^ (marked as ^∗^ in **Figure 4**). Exchange of the residue Y112 to alanine (HrrSY112A) resulted in reduced proteins levels (data not shown), and thus, HrrSY112F was used for further analysis (**Figure 4B**). In comparison with the single mutants, a simultaneous exchange of all three conserved residues (Y^112^-F^115^-F^118^), however, led to no further decrease in target promoter activation (**Figure 4**, marked as ^∗∗^). We speculate that even after complete loss of signal perception, HrrS will still exhibit background kinase activity resulting in the activation of *hmuO* transcription.

Based on the finding for HrrS, we also investigated the second kinase, ChrS, in an analogous experiment. However, here, the plasmid-based overexpression of *chrS* variants was hampered by the fact that ChrS appeared to exhibit a very strong phosphatase activity resulting in weak target promoter activation (pJC1_P*_hrtBA_-eyfp*) upon overproduction. Even for an expression of *chrS* on the same plasmid (pJC1_P*_hrtBA_-eyfp*_P*_chrS_-chrS*), only minor activities could be measured for the reporter (Supplementary Figure S6). Nevertheless, again the exchange of the three residues Y^87^, F^90^, and F^94^ resulted in a significantly lower reporter output. These residues are conserved in the sensor domains of HrrS and ChrS in *C. glutamicum*. However, because of the very low reporter output, the findings for ChrS must be viewed critically.

### HrrS^Y 112A-F115A-F118A^ Shows Impaired Heme-Binding Properties

**FIGURE 5:**
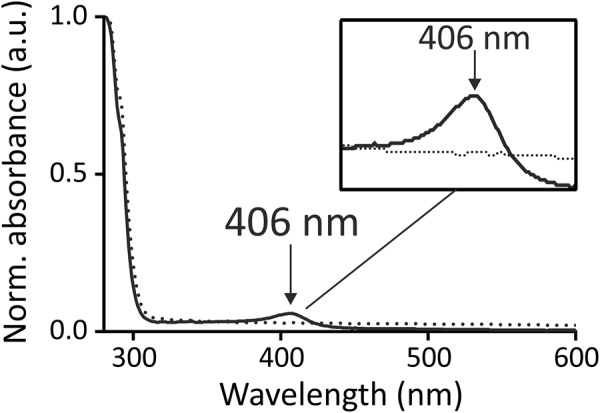
Heme co-purifies together with wild type HrrS (solid line) but not with the triple mutant HrrS^Y 112A-F115A-F118A^ (dotted line). HrrS (solid line) and HrrS^Y 112A-F115A-F118A^ (dotted line) were purified from *E. coli* (BL21) cells as described in the Section “Materials and Methods.” The cells were grown for 16 h, expressing the kinases. No additional heme/hemin was added to the medium or lysate. The purified protein (12 μM) was conducted to UV-vis spectroscopy in a range from 280 to 600 nm. Both spectra were normalized to the absorption of the proteins (at 280 nm) and background absorption of buffer with micelles was subtracted from both spectra. An arrow indicates a small soret peak at 406 nm in the spectrum of wild type HrrS (solid line) which is not observable in the triple mutant (dotted line). The box shows a zoom into the peak area.

In the following, the role of the three aromatic amino acids, as putative heme-binding residues in HrrS, was further investigated in *in vitro* hemin-binding assays. For this purpose, wild type protein as well as the triple mutant HrrS^Y 112A-F115A-F118A^ were overproduced in *E. coli* BL21 and subsequently solubilized and purified as described in the Section “Materials and Methods.” Upon analysis of 12 μM of both proteins (no addition of heme) via UV-vis spectroscopy (280–600 nm) and normalization to the protein absorption (Abs_280_), HrrS consistently showed a soret band at 406 nm which was lacking for the triple mutant (**Figure 5**). This peak corresponds to small amounts of heme co-purified from the *E. coli* extracts. Remarkably, this peak was not observed for the HrrS^Y 112A-F115A-F118A^ triple mutant protein providing first evidence that heme binding of HrrS was impaired by the exchange of these residues (**Figure 5**). Also for the single HrrS mutants (HrrS^Y 112A^, HrrS^F115A^, and HrrS^F118A^), ChrS, and the ChrS^Y 87A-F90A-F94A^ variant, no co-purification was observed either (Supplementary Figure S7).

**FIGURE 6:**
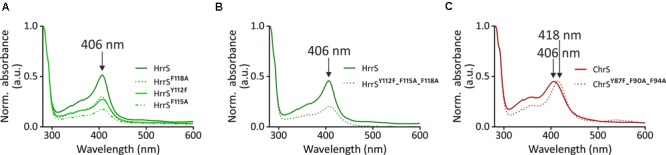
Heme-binding studies with single- and triple mutants of HrrS and ChrS. For all samples, 50 μM hemin was added to the cleared *E. coli* BL21 (DE3) lysate of cells overproducing either HrrS, one of the three HrrS single mutants **(A)** or the HrrS triple mutant **(B)**. Additionally, ChrS and ChrS^Y 87A-F90A-F94A^ were analyzed in an analog experiment **(C)**. After incubation of the lysate with 50 μM hemin for 1 h, ultracentrifugation, solubilization, and purification were carried out as described in the Section “Materials and Methods.” Purified protein samples (12 μM) were conducted to UV-vis spectroscopy and the resulting spectra were normalized to the absorption of the proteins at 280 nm. Background absorption of buffer with DDM micelles was subtracted.

However, only a small fraction of the purified HrrS appeared to be in the heme-bound state. To enhance this effect, 50 μM hemin was added to the cleared *E. coli* lysate prior to protein purification. The lysate was incubated for 1 h at 4°C and subsequently, purification was carried out as described above. After this reconstitution, a significantly increased peak was observed for wild type HrrS after the purification, and now, even the single mutants or the triple mutant HrrS^Y 112A-F115A-F118A^ were co-purified with small amounts of heme/hemin (**Figure 6**). In contrast to the wild type protein, however, the binding was strongly reduced. Similar to the alanine scanning, no clear difference between the single (**Figure 6A**) and the triple mutants (**Figure 6B**) was noticeable, suggesting that all three residues are equally important for the formation of the heme-protein interface. *In vitro* autophosphorylation of HrrS and the triple mutant HrrS^Y 112A-F115A-F118A^ indicated that both proteins are correctly folded and enzymatically active (Supplementary Figure S8).

Analogous experiments were performed for ChrS and the triple mutant ChrS^Y 87A-F90A-F94A^. Both proteins were not purified with bound heme from *E. coli* membranes (Supplementary Figure S7). However, upon addition of hemin to the cleared lysate prior to purification a significant soret band at 406 nm emerged for ChrS demonstrating the ability of this protein to bind heme *in vitro* (**Figure 6C**). Interestingly, for ChrS^Y 87A-F90A-F94A^, the peak volume was not reduced but showed a significant red shift from 406 to 418 nm. Thus, heme binding of ChrS^Y 87A-F90A-F94A^ is somehow affected but obviously, the heme-protein interface differs from HrrS where exchange of the same conserved triplet (HrrS^Y 112A-F115A-F118A^) almost abolished heme binding in the homologous HK.

## Discussion

Bacterial heme homeostasis relies on complex regulatory networks integrating iron- and heme availability to harmonize intracellular processes involved in heme degradation (e.g., HmuO), export (HrtBA), biosynthesis, and heme-containing proteins (Andrews et al., 2003; Yasmin et al., 2011). The Gram-positive soil bacterium *C. glutamicum*, remarkably, invests 2 out of 13 TCS for the maintenance of heme homeostasis (Frunzke et al., 2011; Bott and Brocker, 2012; Heyer et al., 2012). Information on iron availability is integrated into the network via the global iron-dependent regulator DtxR, which represses both the *hmuO* gene as well as *hrrA* encoding the RR of the HrrSA “heme utilization system” (Wennerhold and Bott, 2006). This overall network topology is conserved between the non-pathogenic soil bacterium *C. glutamicum* and the prominent pathogen *C. diphtheriae* highlighting that heme regulatory processes are not restricted to pathogens of vertebrates but represent a significant fitness trait of many bacterial species in the battle for iron.

In this study, we demonstrated that the two HKs of the TCS, HrrSA, and ChrSA, bind heme in a 1:1 stoichiometry per HK monomer. This is in agreement with previous studies reporting the activation of HrrSA and ChrSA target gene expression (e.g., *hmuO* and *hrtBA*, respectively) in response to heme availability (Bibb et al., 2007; Bibb and Schmitt, 2010; Frunzke et al., 2011; Heyer et al., 2012; Hentschel et al., 2014; Burgos and Schmitt, 2016).

**FIGURE 7:**
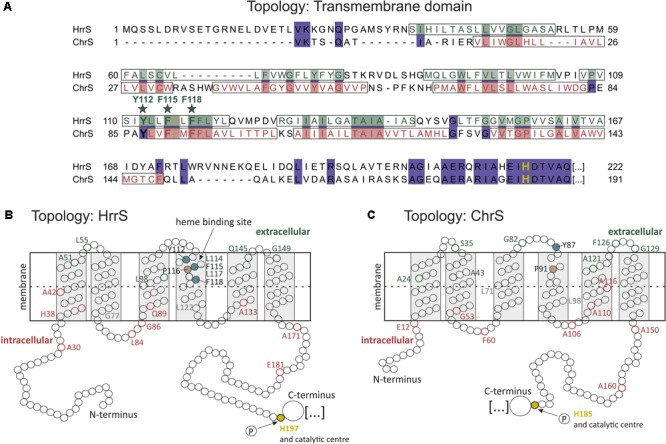
Topology model of the N-terminal transmembrane domain of the two HKs HrrS and ChrS. **(A)** Sequence alignment of the N-terminal parts of HrrS and ChrS was generated using ClustalW, and the transmembrane helices indicated are based on predictions and in agreement with our PhoA/LacZ-Screening. **(B,C)** Scheme of the topology of HrrS and ChrS. Circles represent single amino acids, red indicates high LacZ-activity of the fusion protein (intracellular localization), and green high PhoA activity (extracellular localization). The three putative heme-binding residues of HrrS, Y112, F115, and F118, are indicated as dark green circles in helix number four **(B)**.

For both kinases, heme binding occurs at an N-terminal sensing domain which is embedded in the cytoplasmic membrane via six TMHs connected by only small loop regions (**Figure 7A**). Although our studies revealed a similar membrane topology, the sequence conservation of the sensor domain is rather minor (8.5% sequence identity). This is in line with the finding that heme recognition differs between the systems. Whereas exchange of three conserved aromatic residues (tyrosine 112 and the phenylalanine residues 115 and 118) almost abolished heme binding of HrrS, exchange of these amino acids in the sensor domain of ChrS only resulted in a red shift of the soret band toward an absorption maximum at 418 nm. Differences in heme perception are also reflected by the profiling assay demonstrating a much more sensitive response of the ChrS kinase to heme as a stimulus (**Figure 2**). The central iron atom of heme can either be bound in a penta- or hexa-coordinated manner (Brewitz et al., 2017). The position of the soret band of heme-binding proteins is significantly influenced by the chemical nature of the amino acid ligands (Vickery et al., 1976). Because of this, one can speculate that an altered set of ligands with different chemical properties participates in heme binding of the triple mutant ChrS^Y 87A-F90A-F94A^, resulting in a red shift of the soret band. This set of alternative ligands seems not to be present in HrrS, however. Based on these findings, we cannot exclude that the triplet of conserved aromatic residues is involved in ChrS heme binding as well.

Sequence analysis of ChrS/HrrS paralogs revealed that the three aromatic residues are highly conserved in different *Corynebacteriaceae* species (Supplementary Figure S3). In their recent study, Li et al. (2011) analyzed the top five amino acids with high frequencies in heme-binding pockets and concluded that cysteine, histidine, methionine as well as tyrosine and phenylalanine are the most prominent residues in such interaction centers. Attributed to the chemical versatility of the heme molecule, different heme-protein interactions are possible (Brewitz et al., 2017). Whereas amino acids may act as axial ligands of the central iron atom via sulfur, nitrogen or oxygen donor atoms (cysteine, histidine, lysine, and tyrosine, respectively), π-stacking interactions via aromatic residues (phenylalanine, tyrosine, or tryptophan) may also contribute to the heme interface. For HrrS, we identified three aromatic residues (Y^112^, F^115^, and F^118^), which upon exchange, led to a reduced reporter output in response to heme. Remarkably, an exchange of Y112 to phenylalanine (lacking the hydroxyl group of tyrosine) led to a drastically reduced heme binding (**Figures 5, 6**). We therefore speculate that the tyrosine 112 may act as an axial ligand of the central iron atom. Also in NEAT (NEAr Transporter) proteins, several well-studied cases confirmed single tyrosine residues coordinating the central metal ion of the heme molecule (Grigg et al., 2007; Sharp et al., 2007; Villareal et al., 2008).

While Y^112^ is concluded to be crucial for the interaction with the iron atom, the phenylalanine residues F^115^ and F^118^ might be important for the interface through aromatic stacking interactions with the porphyrin ring of heme (Schneider et al., 2007; Smith et al., 2010; Li et al., 2011). Interestingly, a single proline residue (P^116^) is located between the two phenylalanine residues and is, together with the YFF motif, conserved between HrrS and ChrS (**Figure 6A**). This amino acid might be involved in shaping the heme interface by kinking the α-helix number four (von Heijne, 1991) thereby creating a heme-binding pocket consisting of the three residues Y^112^-F^115^-Y^118^ (**Figure 7B**). This effect has been demonstrated for the cysteine-proline (CP) dipeptide motif representing a prominent heme-regulatory motif (Lathrop and Timko, 1993).

In *C. diphtheriae*, the ChrS membrane topology was predicted using the two tools TMpred and DAS and experimentally analyzed via six PhoA/LacZ fusions (Bibb and Schmitt, 2010). Comparable to HrrS and ChrS in *C. glutamicum*, six TMHs were postulated (**Figures 7B,C**). In this study, alanine scanning of the N-terminus of this homologous protein revealed several kinase mutants with inability to activate the target operon *hrtBA*. A conserved tyrosine residue in TMH number two (Y^61^) was postulated as a prime candidate for heme binding (Bibb and Schmitt, 2010). This amino acid Y^61^ in ChrS (*C. diphtheriae*) corresponds to Y^74^ in HrrS (*C. glutamicum*, see Supplementary Figure S3). However, in our study the exchange of this residue to alanine had no significant effect (**Figure 4**). In contrast, the exchange of phenylalanine residue 114 to alanine in *C. diphtheriae* ChrS (TMH number four) led to a 40% decreased P*_hrtBA_* activity. Interestingly, this residue is conserved between most *Corynebacteriaceae* and as supporting evidence phenylalanine F^118^ is part of our postulated Y^112^-F^115^-F^118^ heme-protein interface in HrrS, suggesting a conserved function and mode of interaction.

As a further heme-responsive TCS, Stauff and Skaar (2009a,b) characterized the conserved HssRS of *Staphylococcus aureus* and *Bacillus anthracis* and predicted an N-terminal periplasmic sensing domain flanked by two transmembrane helices. Their findings indicate that the mode of signal perception of HssS (*S. aureus* and *B. anthracis*) vastly differs from HrrS/ChrS from *Corynebacterium* species. Exchange of several residues in the extracellular part of *B. antracis* HssS did not lead to the identification of the heme-binding site, but the fact, that the sensing domain is flanked by two helices led to the prediction, that this protein detects a signal located either in the periplasm or within the membrane itself, similar to HrrS (Stauff and Skaar, 2009a). As in our study, the exchange of residues likely not involved in heme binding (such as arginine or lysine) showed reduced phenotype complementation and led to the speculation that this domain might be more involved in signal transduction than previously expected.

In summary, this study provides evidence for a direct sensing of heme via the N-terminal sensor domain of the HKs ChrS and HrrS in *C. glutamicum*. Although the particular binding pocket may differ between the proteins, they represent two further examples of proteins involved in transient heme-sensing of bacteria. Considering the versatile composition of heme–protein interfaces in nature, the molecular and structural analysis of heme proteins provides an important basis for the prediction of novel domains potentially involved in heme sensing.

## Author Contributions

MK, ED, and JF conceived the study; MK, ED, and CG performed the experiments; MK and ED analyzed the data; and MK and JF wrote the manuscript.

## Conflict of Interest Statement

The authors declare that the research was conducted in the absence of any commercial or financial relationships that could be construed as a potential conflict of interest.
