# Supplementary material for: Membrane Topology and Heme Binding of the Histidine Kinases HrrS and ChrS in Corynebacterium glutamicum
Source: Front Microbiol. 2018 Feb 9;9:183. doi: 10.3389/fmicb.2018.00183 (PMC5812335; doi:10.3389/fmicb.2018.00183)
Supplement: Supplementary file 1 [file Table_1.PDF]

Supplementary Material

Membrane topology and heme binding of the histidine kinases HrrS and ChrS in Corynebacterium glutamicum

Marc Keppel[1], Eva Davoudi[1], Cornelia Gätgens[1], and Julia Frunzke[1]*

[1]Institute of Bio- und Geosciences, IBG-1: Biotechnology, Forschungszentrum Jülich,52425 Jülich, Germany

*Corresponding author: Julia Frunzke; Email: j.frunzke@fz-juelich.de; Phone: +49 2461 615430

1 Supplementary Data

2 Supplementary Figures and Tables

2.1 Supplementary Figures

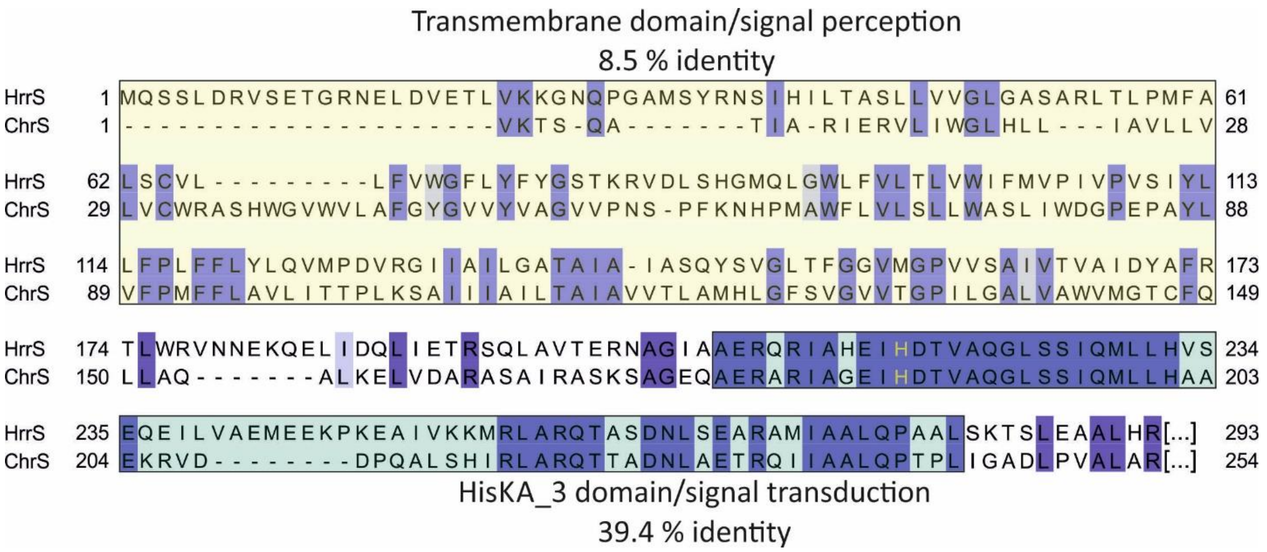

Supplementary Figure S1 - Amino acid sequence alignment of the histidine kinases HrrS and ChrS of *C. glutamicum*. The alignment was performed using Clustal Omega and jalview. Sequences were obtained from the NCBI database (https://blast.ncbi.nlm.nih.gov).

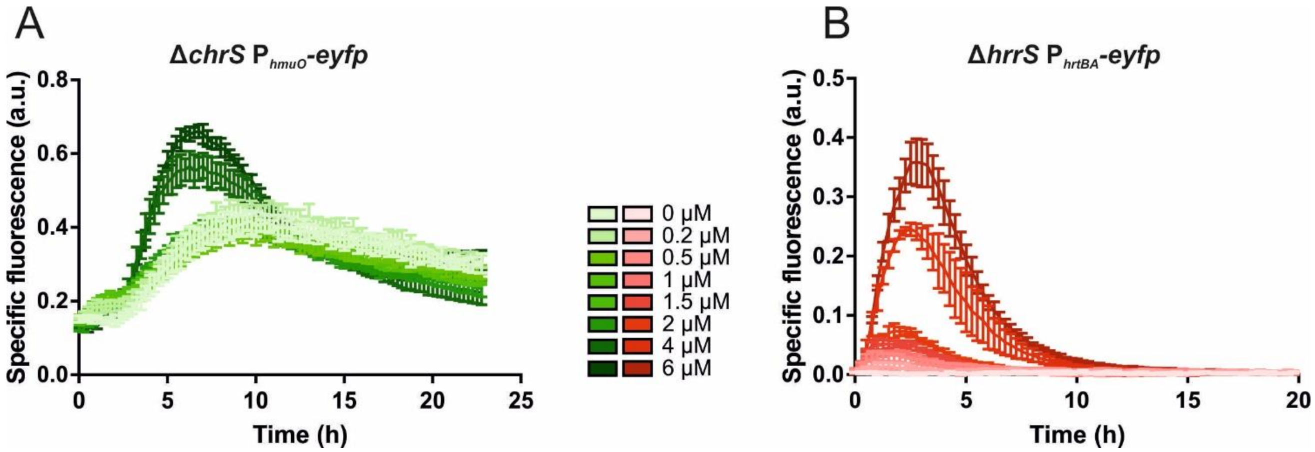

Supplementary Figure S2 - Promoter profiling in Δ*hrrS* and Δ*chrS* deletion mutants. The *C. glutamicum* mutant Δ*chrS* or Δ*hrrS* were transformed with the target gene reporter pJC1_P$_{hmuO}$-*eyfp* or pJC1_P$_{hrtBA}$-*eyfp*, respectively. Cells were cultivated in a microbioreactor system (Biolector) in CGXII minimal medium with 2% (w/v) glucose containing 0-6 µM hemin. The specific fluorescence was referenced against the reporter output of strains showing background level of the particular reporter construct as described previously (Δ*hrrSA* / pJC1-P$_{hmuO}$-*eyfp* and Δ*chrSA* / pJC1-P$_{hrtBA}$-*eyfp* (Hentschel et al., 2014)).

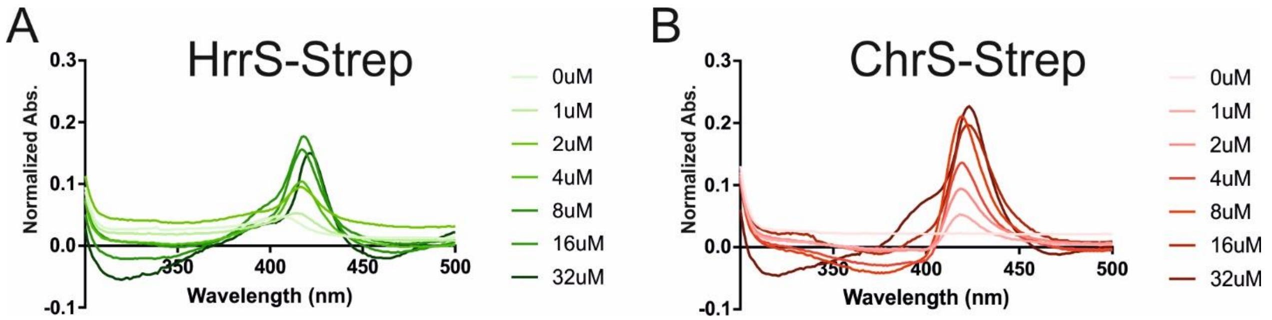

Supplementary Figure S3 - UV-Vis analysis of the heme binding properties of HrrS and ChrS. For hemin binding assays, different amounts of hemin were titrated to 10 µM of purified HrrS or ChrS protein to a final concentration of 0, 2, 4, 8, 16 and 32 µM. The mixture was incubated for 5 min at RT and then analyzed by UV-visual spectroscopy. The resulting absorption was referenced against the absorption of buffer containing only DDM micelles without protein.

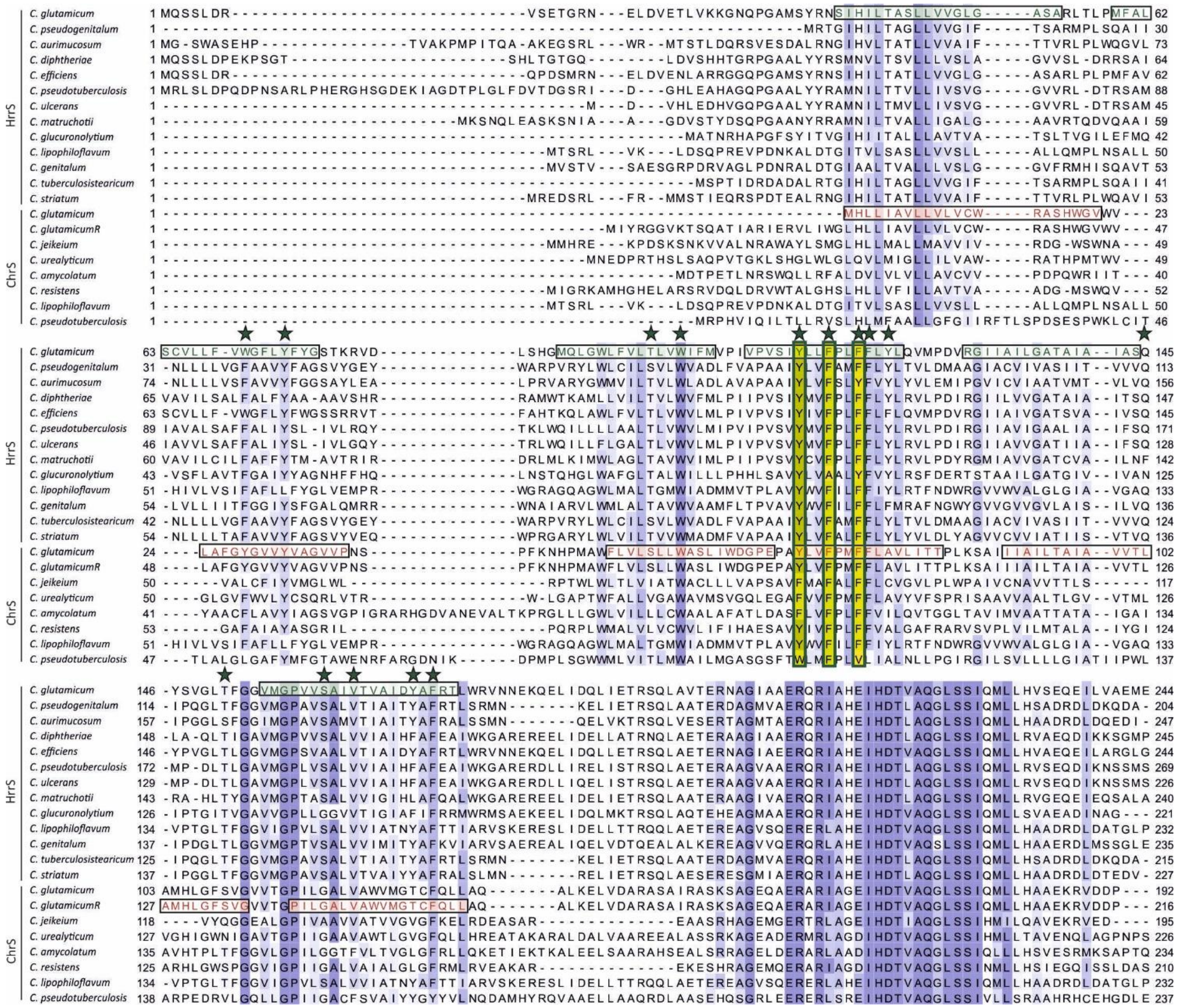

Supplementary Figure S4 - Sequence alignment of HrrS (top) and ChrS (bottom) of different *Corynebacteriaceae*. Alignment was performed using Clustal Omega and jalview. Sequences were obtained from the NCBI database (https://blast.ncbi.nlm.nih.gov). Asterisks mark amino acids analyzed by alanine-scanning of HrrS (Figure 4) and ChrS (Figure S6). The conserved trio (Y112-F115-F118, in *C. glutamicum* HrrS) of aromatic residues are highlighted in yellow. Exchange of the respective residues resulted in an almost abolished heme-binding of HrrS *in vitro* (Figure 5 and 6).

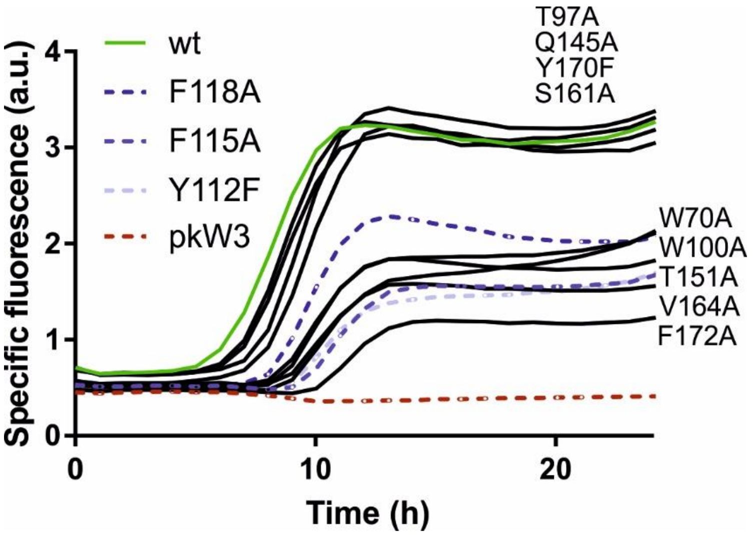

Supplementary Figure S5 - Alanine scanning of the transmembrane domain of HrrS reveals putative heme binding residues. The *C. glutamicum* mutant strain Δ*hrrS*Δ*chrS* was transformed with the target gene reporter pECXC99E_P$_{hmuO}$-*eyfp* and the pKW3 plasmid either containing wild type *hrrS* under its native promoter (wt) or one of fifteen *hrrS* variants, encoding the histidine kinase with a single amino acid exchange. All proteins contained a C-terminal FLAG tag fusion for western blot analysis. Cells were cultivated in a microbioreactor system (Biolector) in CGXII minimal medium with 2% (w/v) glucose containing 2.5 µM hemin.

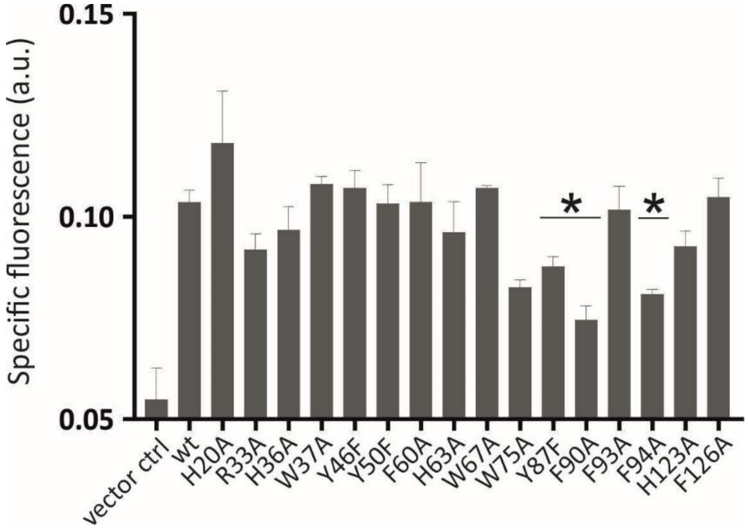

Supplementary Figure S6 - Alanine scanning of the transmembrane domain of ChrS revealed amino acid residues putatively involved in heme binding. A *C. glutamicum* Δ*hrrS*Δ*chrS* mutant strain was transformed with the target gene reporter pJC1_PhrtBA-eypf (kanamycin resistance) and either containing wild type *chrS* under native promoter (labeled wt) or one of fifteen *chrS* variants, encoding the histidine kinase with a single amino acid exchange. Cells were cultivated in the BioLector system in CGXII minimal medium with 2% (w/v) glucose containing 2.5 µM hemin. The peak specific fluorescence (fluorescence signal per backscatter signal, given in arbitrary units, a.u.) after 4 h is shown in the graph. The data represent average values of three independent biological replicates.

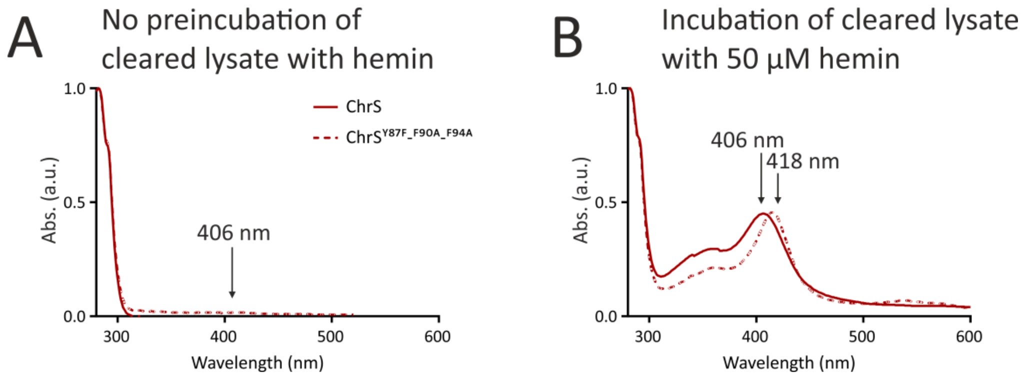

Supplementary Figure S7 - Heme/hemin binding properties of purified ChrS and ChrSY87A-F90A-F94A. ChrS is not co-purified together with heme from the *E. coli* lysate, but shows significant hemin binding properties after incubation with 50 µM hemin in the *E. coli* crude extract. The membrane proteins were purified by the means of a C-terminal Strep-tag and analyzed the UV/Visible double beam spectrophotometer UV-1601 PC (Shimadzu, Kyoto, Japan).

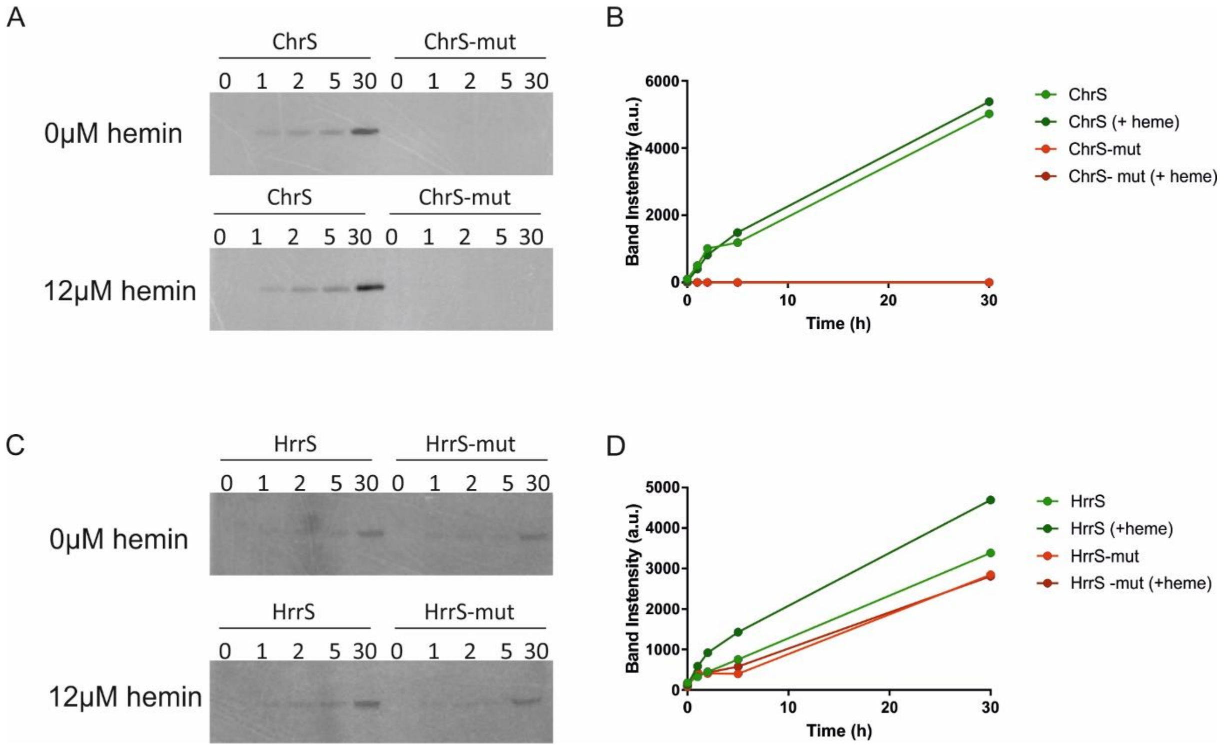

Supplementary Figure S8 - Autophosphorylation of full length histidine kinases HrrS-Strep and ChrS-Strep. Immediately after purification, both kinases were incubated with 0.25 µM[γ-^{33}P]-ATP (10 mCi/ml; PerkinElmer, USA) mixed with 80 µM non-radioactive ATP. The mixture was incubated for up to 30 min and at different time points aliquots were removed and the phosphorylation state was analyzed on storage phosphor imaging films (Fuji Photo Film Co., Tokyo, Japan) and with a Typhoon Trio Scanner (GE Healthcare, Germany).

12 µM of purified HrrS/HrrSY112F-F115A-F118A (C) and ChrS/ChrSY87A-F90A-F94A (A) either with (top) or without (bottom) addition of hemin. B and D: Band intensities were analyzed with Fiji ImageJ (https://fiji.sc/).

2.2 Supplementary Tables

Supplementary Table S1. Bacterial strains and plasmids.

Strain or plasmid	Relevant characteristics	Source or reference
	E. coli	
DH5α	*fhuA2 lac(del)U169 phoA glnV44 Φ80' lacZ(del)M15 gyrA96 recA1 relA1 endA1 thi-1 hsdR17*; for general cloning purposes	Invitrogen

TG-1	K-12 *glnV44 thi-1 Δ(lac-proAB) Δ(mcrB-hsdSM)5,* ($r_K^-m_K^-$) F′ [*traD36 proAB*$^+$ *lacI*^{q} *lacZΔM15*]; for *phoA*/*lacZ* assays	Lucigen
BL21(DE3)	B F$^-$ *ompT gal dcm lon hsdS*$_B$($r_B^-m_B^-$) λ(DE3 [*lacI lacUV5-T7p07 ind1 sam7 nin5*]) [*malB*$^+$]$_{K-12}$($λ^S$); overexpression of membrane proteins.	(Studier & Moffatt, 1986)
	C. glutamicum	
ATCC 13032	Wildtype strain *Corynebacterium glutamicum*	(Kinoshita *et al.*, 2004)
ATCC 13032 Δ*hrrS*Δ*chrS*	Deletion mutant of the two genes encoding the HKs HrrS and ChrS	(Hentschel *et al.*, 2014)
ATCC 13032 Δ*hrrS*	Deletion mutant of the open reading frame (orf) encoding the HK HrrS	(Hentschel *et al.*, 2014)
ATCC 13032 Δ*chrS*	Deletion mutant of the orf encoding the HK ChrS	(Hentschel *et al.*, 2014)
	**Plasmids**	
pJC1_P$_{hmuO}$-*eyfp*	Target gene reporter for the HrrSA system	(Heyer *et al.*, 2012)
pJC1_P$_{hrtBA}$-*eyfp*	Target gene reporter for the ChrSA system	(Heyer *et al.*, 2012)
pEC-XC99E_ P$_{hmuO}$-*eyfp*	Alternative target gene reporter for the HrrSA system with chloramphenicol resistance. Amplification of the P$_{hmuO}$-*eyfp* fragment (Oligo 42 and 43) and ligation into the pEC-XC99E vector backbone.	This work
pT7-5-*phoA*	Expression plasmid for *E. coli*, carries *phoA* as 3' extension behind the MCS, kindly supplied by the laboratories of G. Unden	(Bauer *et al.*, 2011)
pT7-5-*lacZ*	Expression plasmid for *E. coli*, carries *lacZ* as 3' extension behind the MCS	(Bauer *et al.*, 2011)
pT7-5_*hrrS-phoA* (*hrrS*A30-*hrrS*E424)	The pT7-5 plasmid was digested with *BamH*I and *Nhe*I and 20 different truncated versions of *hrrS* were amplified (Oligo 1-21), digested with the	This work

	same restriction enzymes and ligated into the linearized plasmid.	
pT7-5_*hrrS-lacZ* (*hrrS*A30-*hrrS*E424)	See construction of pT7-5_*hrrS-phoA*	This work
pT7-5_*chrS-phoA* (*chrS*E12-*chrS*A160)	The pT7-5 plasmid was digested with *BamH*I and *Nhe*I and 20 different truncated versions of *chrS* were amplified (Oligo 22–41), digested with the same restriction enzymes and ligated into the linearized plasmid	This work
pT7-5_*chrS-lacZ* (*chrS*E12-*chrS*A160)	See construction of pT7-5_*chrS-phoA*	This work
pET24b-*hrrS*-Cs*trep*	IPTG inducible expression plasmid for the overexpression of *hrrS-strep* (full-length) in *E. coli* BL21. Amplification of *hrrS* wt from chromosomal DNA with the oligonucleotides 44 and 45, ligation into pET24b *via* Gibson assembly.	This work
pET24b-*chrS*-Cs*trep*	IPTG inducible expression plasmid for the overexpression of *chrS-strep* (full-length) in *E. coli* BL21. Amplification of *chrS* wt from chromosomal DNA with the oligonucleotides 46 and 47, ligation into pET24b *via* Gibson assembly.	This work
pET24b-*hrrSY112F-F115A-F118A*-Cs*trep*	Single mutations were introduced into the plasmid *via* the "quik-change-lightning" kit (Agilent Genomics, Santa Clara, United States) and for the triple mutations additional rounds of mutagenesis were performed	This work
pET24b-*chrSY87F-F90A-F94A*-Cs*trep*	Single mutations were introduced into the plasmid *via* the "quik-change-lightning" kit (Agilent Genomics, Santa Clara, United States) and for the triple mutations additional rounds of mutagenesis were performed	This work
pKW3	Expression plasmid; containing a high copy number ori for *E. coli* and a low copy number ori for *C. glutamicum*	(Eggeling *et al.*, 1998)

pKW3_*hrrS-flag*	*hrrS* including native promoter (~300 Bp upstream of ATG) was amplified with the oligonucleotides 106 and 107 and cut with *EcoR*I and *BamH*I. pKW3 was linearized with *EcoR*I and *BamH*I and the PCR fragment ligated to generate the circular plasmid.	This work
pKW3_*hrrS-flag* (*hrrSW70A-F172A)*	16 single mutations were introduced into the pKW3_*hrrS-flag* plasmid to generate 16 different single mutation expression plasmids. Mutations were introduced into the plasmid *via* the "quik-change-lightning" kit (Agilent Genomics, Santa Clara, USA) and the oligonucleotides 48 - 79 as described in material and methods.	This work
pJC1_P_{hrtBA}-*eyfp_chrS-flag* (*chrSH20A-F126A*)	16 single mutations were introduced into the pJC1_P_{hrtBA}-*eyfp_chrS-flag* plasmid to generate 16 different single mutation expression plasmids. Mutations were introduced into the plasmid *via* the "quik-change-lightning" kit (Agilent Genomics, Santa Clara, USA).	This work

Supplementary Table S2. Oligonucleotides used in this study. Restriction sites are underlined.

#	Oligonucleotide	Sequence
		**PhoA/LacZ Screening**
**1**	hrrS_phoA-lacZ_fw	GCGC**<u>GGATCC</u>**ATGCAGTCAAGCCTAGATCG
**2**	hrrS-A30_phoA/lacZ_rv	GCGC**<u>GCTAGC</u>**CGCGCCCGGTTGATTCCCCTTC
**3**	hrrS-H38_phoA/lacZ_rv	GCGC**<u>GCTAGC</u>**GTGGATACTGTTGCGATAGCTC
**4**	hrrS-A42_phoA/lacZ_rv	GCGC**<u>GCTAGC</u>**GGCTGTCAAAATGTGGATAC
**5**	hrrS-A51_phoA/lacZ_rv	GCGC**<u>GCTAGC</u>**AGCTCCCAACCCCACGACCAG
**6**	hrrS-L55_phoA/lacZ_rv	GCGC**<u>GCTAGC</u>**CAGGCGGGCGGAAGCTCCCAAC
**7**	hrrS-G77_phoA/lacZ_rv	GCGC**<u>GCTAGC</u>**TCCATAGAAGTACAGAAAACCCC
**8**	hrrS-L84_phoA/lacZ_rv	GCGC**<u>GCTAGC</u>**CAAATCTACGCGTTTGGTTGATC
**9**	hrrS-G87_phoA/lacZ_rv	GCGC**<u>GCTAGC</u>**GCCGTGGCTCAAATCTACGC
**10**	hrrS-Q89_phoA/lacZ_rv	GCGC**<u>GCTAGC</u>**CTGCATGCCGTGGCTCAAATC
**11**	hrrS-L96_phoA/lacZ_rv	GCGC**<u>GCTAGC</u>**CAGCACAAACAGCCAGCCCAG
**12**	hrrS-L114_phoA/lacZ_rv	GCGC**<u>GCTAGC</u>**CAGCAGATAAATGGACACGG
**13**	hrrS-L117_phoA/lacZ_rv	GCGC**<u>GCTAGC</u>**CAGCGGAACAGCAGATAAATGG
**14**	hrrS-L122_phoA/lacZ_rv	GCGC**<u>GCTAGC</u>**TAGATAGAGGAAAAACAGCG
**15**	hrrS-A133_phoA/lacZ_rv	GCGC**<u>GCTAGC</u>**CGCAATAATGCCTCTCACGTC
**16**	hrrS-A143_phoA/lacZ_rv	GCGC**<u>GCTAGC</u>**CCCCACGGAATACTGGCTG

**17**	hrrS-Q145_phoA/lacZ_rv	GCGC**GCTAGC**CTGGCTGGCAATCGCAATCG
**18**	hrrS-A171_phoA/lacZ_rv	GCGC**GCTAGC**CGCGTAATCAATAGCCACGG
**19**	hrrS-E181_phoA/lacZ_rv	GCGC**GCTAGC**TTCATTATTCACCCGCCACAAC
**20**	hrrS-H214_phoA/lacZ_rv	GCGC**GCTAGC**ATGCGCAATACGTTGACGTTC
**21**	hrrS-E424_phoA/lacZ_rv	GCGC**GCTAGC**CTCATCGTCAGTTGGAGAAC
**22**	chrS-E18_phoA/lacZ_rv	GCGC**GCTAGCC**TCAATTCGGGCGATGGTC
**23**	chrS-A30_phoA/lacZ_rv	GCGC**GCTAGC**GGCAATGAGTAAATGCAATC
**24**	chrS-S41_phoA/lacZ_rv	GCGC**GCTAGC**GCTGGCACGCCAACACACCAAC
**25**	chrS-A49_phoA/lacZ_rv	GCGC**GCTAGC**AGCGAGCACCCACACACCCC
**26**	chrS-G59_phoA/lacZ_rv	GCGC**GCTAGC**ACCCGCCACATAAACCACG
**27**	chrS-F66_phoA/lacZ_rv	GCGC**GCTAGC**AAACGGCGAATTCGGGACCAC
**28**	chrS-L77_phoA/lacZ_rv	GCGC**GCTAGC**CAGCACAAGAAACCACGCCATAG
**29**	chrS-G88_phoA/lacZ_rv	GCGC**GCTAGC**TCCATCCCAAATCAGGCTCG
**30**	chrS-F96_phoA/lacZ_rv	GCGC**GCTAGC**AAACACCAAATACGCAGGCTCC
**31**	chrS-F100_phoA/lacZ_rv	GCGC**GCTAGC**GAAAAACATCGGAAACACC
**32**	chrS-F104_phoA/lacZ_rv	GCGC**GCTAGC**CAACACTGCGAGGAAAAACATC
**33**	chrS-K110_phoA/lacZ_rv	GCGC**GCTAGC**TTTCAGCGGTGTCGTGATCAAC
**34**	chrS-A112_phoA/lacZ_rv	GCGC**GCTAGC**CGCGGATTTCAGCGGTGTCG
**35**	chrS-A116_phoA/lacZ_rv	GCGC**GCTAGC**TGCAATGATGATCGCGGATTTCAG
**36**	chrS-A122_phoA/lacZ_rv	GCGC**GCTAGC**CGCGATCGCCGTCAGTATTG
**37**	chrS-A127_phoA/lacZ_rv	GCGC**GCTAGC**AGCCAACGTAACCACCGCGATC
**38**	chrS-F132_phoA/lacZ_rv	GCGC**GCTAGC**AAACCCCAGGTGCATAGCCAAC
**39**	chrS-G135_phoA/lacZ_rv	GCGC**GCTAGC**GCCAACAGAAAACCCCAGGTG
**40**	chrS-L156_phoA/lacZ_rv	GCGC**GCTAGC**TAACTGAAAACACGTACCC
**41**	chrS-A167_phoA/lacZ_rv	GCGC**GCTAGC**TGCGTCGACAAGCTCCTTTAAG
	**pECXC99E reporter**	
**42**	PhmuO_fw	GCGC**CATATG**CTAGCGAAGTTCTTGAAGTG
**43**	PhmuO_rv	GCGC**GTCGAC**TTATCTAGACTTGTACAGCTCG
	**Overexpression plasmids HrrS/ChrS-Strep**	
**44**	hrrS_Cstrep_fw	ACTTTAAGAAGGAGATATACATATGATGCAGTCAAGCCTAGATCG
**45**	hrrS_Cstrep_rv	CCTGAAAATACAGGTTCTCGCTAGCCTCATCGTCAGTTGGAGAAC
**46**	chrS_Cstrep_fw	ACTTTAAGAAGGAGATATACATATGGTGAAAACTAGCCAAGCGACC
**47**	chrS_Cstrep_rv	CCTGAAAATACAGGTTCTCGCTAGCCTTATCTTGGTCCTTTTGTGG
**Point mutations *hrrS/chrS***		
**48**	hrrS-W70_A_fw	ATAGAAGTACAGAAAACCCGCCACAAACAACAGCACGCAC
**49**	hrrS-W70_A_rv	GTGCGTGCTGTTGTTTGTGGCGGGTTTTCTGTACTTCTAT
**50**	hrrS-Y74_A_fw	CGTTTGGTTGATCCATAGAAGGCCAGAAAACCCCACACAAACAA
**51**	hrrS-Y74_A_rv	TTGTTTGTGTGGGGTTTTCTGGCCTTCTATGGATCAACCAAACG
**52**	hrrS-T97A-fw	CTGGCTGTTTGTGCTGGCGCTGGTGTGGATTTT
**53**	hrrS-T97A-rv	AAAATCCACACCAGCGCCAGCACAAACAGCCAG
**54**	hrrS-W100_A_fw	GATCGGCACCATAAAAATCGCCACCAGCGTCAGCACAAAC
**55**	hrrS-W100_A_rv	GTTTGTGCTGACGCTGGTGGCGATTTTTATGGTGCCGATC
**56**	hrrS-Y112A_fw	GCGGGAACAGCAGAGCAATGGACACGGGCACGATC
**57**	hrrS-Y112A_rv	GATCGTGCCCGTGTCCATTGCTCTGCTGTTCCCGC

**58**	hrrS-Y112F-fw	CGATCGTGCCCGTGTCCATTTTTCTGCTGTTCC
**59**	hrrS-Y112F-rv	GGAACAGCAGAAAAATGGACACGGGCACGATCG
**60**	hrrS-F115A-fw	CGTGTCCATTTATCTGCTGGCCCCGCTGTTTTTCCTCTAT
**61**	hrrS-F115A-rv	ATAGAGGAAAAACAGCGGGGCCAGCAGATAAATGGACACG
**62**	hrrS-F118_A_fw	CACCTGTAGATAGAGGAAAGCCAGCGGGAACAGCAGATAA
**63**	hrrS-F118_A_rv	TTATCTGCTGTTCCCGCTGGCTTTCCTCTATCTACAGGTG
**64**	hrrS-F119_A_fw	CACCTGTAGATAGAGGGCAAACAGCGGGAACAGCAGATAA
**65**	hrrS-F119_A_rv	TTATCTGCTGTTCCCGCTGTTTGCCCTCTATCTACAGGTG
**66**	hrrs-Y121_A_fw	GTCAGGCATCACCTGTAGAGCGAGGAAAAACAGCGGGAAC
**67**	hrrs-Y121_A_rv	GTTCCCGCTGTTTTTCCTCGCTCTACAGGTGATGCCTGAC
**68**	hrrS-Q145A-fw	GATTGCGATTGCCAGCGCGTATTCCGTGGGGTTG
**69**	hrrS-Q145A-rv	CAACCCCACGGAATACGCGCTGGCAATCGCAATC
**70**	hrrS-T151A-fw	GTATTCCGTGGGGTTGGCCTTTGGTGGTGTGAT
**71**	hrrS-T151A-rv	ATCACACCACCAAAGGCCAACCCCACGGAATAC
**72**	hrrS-S161A-fw	GGGTCCGGTGGTCGCTGCGATCGTGAC
**73**	hrrS-S161A-rv	GTCACGATCGCAGCGACCACCGGACCC
**74**	hrrS-V164_A_fw	CAATAGCCACGGTCGCGATCGCAGAGACC
**75**	hrrS-V164_A_rv	GGTCTCTGCGATCGCGACCGTGGCTATTG
**76**	hrrS-Y170F-fw	GTGACCGTGGCTATTGATTTCGCGTTCCGC
**77**	hrrS-Y170F-rv	GCGGAACGCGAAATCAATAGCCACGGTCAC
**78**	hrrS-F172A-fw	GGCTATTGATTACGCGGCCCGCACGTTGTGGCGG
**79**	hrrS-F172A-rv	CCGCCACAACGTGCGGGCCGCGTAATCAATAGCC
**80**	chrS-H20A-fw	GAGAGTTCTCATTTGGGGATTGGCTTTACTCATTGCCGTTTTGTTG
**81**	chrS-H20A-rv	CAACAAAACGGCAATGAGTAAAGCCAATCCCCAAATGAGAACTCTC
**82**	chrS-H36A-fw	GTTGGCGTGCCAGCGCTTGGGGTGTGTGGG
**83**	chrS-H36A-rv	CCCACACACCCCAAGCGCTGGCACGCCAAC
**84**	chrS-W37A-fw	TGGCGTGCCAGCCATGCGGGTGTGTGGGT
**85**	chrS-W37A-rv	ACCCACACACCCGCATGGCTGGCACGCCA
**86**	chrS-Y46F-fw	TGGGTGCTCGCTTTTGGCTTTGGCGTGGTT
**87**	chrS-Y46F-rv	AACCACGCCAAAGCCAAAAGCGAGCACCCA
**88**	chrS-F60A-fw	GTGGTCCCGAATTCGCCGGCTAAGAATCACCCTATGGC
**89**	chrS-F60A-rv	GCCATAGGGTGATTCTTAGCCGGCGAATTCGGGACCAC
**90**	chrS-H63A-fw	TCCCGAATTCGCCGTTTAAGAATGCCCCTATGGCGTG
**91**	chrS-H63A-rv	CACGCCATAGGGGCATTCTTAAACGGCGAATTCGGGA
**92**	chrS-W67A-fw	TAAGAATCACCCTATGGCGGCGTTTCTTGTGCTGAGTTTG
**93**	chrS-W67A-rv	CAAACTCAGCACAAGAAACGCCGCCATAGGGTGATTCTTA
**94**	chrS-W75A-fw	CTTGTGCTGAGTTTGTTGGCGGCGAGCCTGATTTGGG
**95**	chrS-W75A-rv	CCCAAATCAGGCTCGCCGCCAACAAACTCAGCACAAG
**96**	chrS-Y87F-fw	GACCGGAGCCTGCGTTTTTGGTGTTTCCGAT
**97**	chrS-Y87F-rv	ATCGGAAACACCAAAAACGCAGGCTCCGGTC
**98**	chrS-F90A-fw	GAGCCTGCGTATTTGGTGGCTCCGATGTTTTTCCTCGC
**99**	chrS-F90A-rv	GCGAGGAAAAACATCGGAGCCACCAAATACGCAGGCTC
**100**	chrS-F93A-fw	CGTATTTGGTGTTTCCGATGGCTTTCCTCGCAGTGTTGATCA

**101**	chrS-F93A-rv	TGATCAACACTGCGAGGAAAGCCATCGGAAACACCAAATACG
**102**	chrS-H123A-fw	GTGGTTACGTTGGCTATGGCCCTGGGGTTTTCTGTTGG
**103**	chrS-H123A-rv	CCAACAGAAAACCCCAGGGCCATAGCCAACGTAACCAC
**104**	chrS-F126A-fw	GGCTATGCACCTGGGGGCTTCTGTTGGCGTTGTC
**105**	chrS-F126A-rv	GACAACGCCAACAGAAGCCCCCAGGTGCATAGCC
		**Generation of pKW3_P*hrrS*-*hrrS-flag***
**106**	P*hrrS*-*hrrS*_fw	TATA**GAATTC**CGGCGACCCAGTCGGTGC
**107**	P*hrrS*-*hrrS*_rv(FLAG)	GCGC**GGATCC**TTACTTGTCGTCATCGTCTTTGTAGTCCTCATCGTCAGTTGGAGAACTTAG

Supplementary Table S3. Number of transmembrane helices (TMHs) predicted by several different online tools. The transmembrane domain of both HKs was analyzed using six different online tools for the prediction of membrane spanning helices: TopPredII (Claros & von Heijne, 1994), TMPred (Hofman & Stoffel, 1993), Hmmtop (Tusnady & Simon, 2001), Minnou polyview (Porollo *et al.*, 2004), CBS TMHMM (Krogh *et al.*, 2001), DAS (Cserzo *et al.*, 1997), Mpex (Snider *et al.*, 2009), TOPCONS (Tsirigos *et al.*, 2015) and Phobius (Kall *et al.*, 2007). TopPredII predicted three and five helices for HrrS and ChrS, respectively, but suggested a forth (or sixth) helix with lower probability. The prediction of Hmmtop and TOPCONS were in line with the experimental data and are highlighted in yellow.
Links: TopPredII [https://bioweb.pasteur.fr/seqanal/interfaces/toppred.html], TMpred [https://www.ch.embnet.org/software/TMPRED_form.html], HMMTOP [www.enzim.hu/hmmtop], Minnou polyview [http://minnou.cchmc.org/], CBS TMHMM [www.cbs.dtu.dk/services/TMHMM/], DAS [tmdas.bioinfo.se/], Mpex [http://blanco.biomol.uci.edu/mpex/], TOPCONS [http://topcons.cbr.su.se] and Phobius [http://phobius.sbc.su.se].

	**HrrS**	**ChrS**
**Program**	**predicted TMHs**	**predicted TMHs**
**TopPredII**	3(4?)	5(6?)
**TMPred**	3	5
**HMMTOP**	6	6
**Minnou polyview**	7	5
**CBS TMHMM**	4	5
**DAS**	5	5
**Mpex**	5	5
**TOPCONS**	6	6
**Phobius**	5	6

$$Miller\ units = 1000 * \frac{Abs420 - 1{,}7 * Abs550}{time\ (\text{min}) * Abs600}$$

Formula S1: Calculation of alkaline phosphatase and β-galactosidase activity in miller units.

3 References

Bauer, J., Fritsch, M. J., Palmer, T. & Unden, G. (2011). Topology and accessibility of the transmembrane helices and the sensory site in the bifunctional transporter DcuB of *Escherichia coli*. *Biochemistry* **50**, 5925-5938.

Claros, M. G. & von Heijne, G. (1994). TopPred II: an improved software for membrane protein structure predictions. *Computer applications in the biosciences : CABIOS* **10**, 685-686.

Cserzo, M., Wallin, E., Simon, I., von Heijne, G. & Elofsson, A. (1997). Prediction of transmembrane alpha-helices in prokaryotic membrane proteins: the dense alignment surface method. *Protein engineering* **10**, 673-676.

Eggeling, L., Oberle, S. & Sahm, H. (1998). Improved L-lysine yield with Corynebacterium glutamicum: use of dapA resulting in increased flux combined with growth limitation. *Applied microbiology and biotechnology* **49**, 24-30.

Hentschel, E., Mack, C., Gatgens, C., Bott, M., Brocker, M. & Frunzke, J. (2014). Phosphatase activity of the histidine kinases ensures pathway specificity of the ChrSA and HrrSA two-component systems in *Corynebacterium glutamicum*. *Molecular microbiology* **92**, 1326-1342.

Heyer, A., Gatgens, C., Hentschel, E., Kalinowski, J., Bott, M. & Frunzke, J. (2012). The two-component system ChrSA is crucial for haem tolerance and interferes with HrrSA in haem-dependent gene regulation in *Corynebacterium glutamicum*. *Microbiology (Reading, England)* **158**, 3020-3031.

Hofman, K. & Stoffel, W. (1993). A database of membrane spanning proteins segments. *Journal of Biological Chemistry* **374**.

Kall, L., Krogh, A. & Sonnhammer, E. L. (2007). Advantages of combined transmembrane topology and signal peptide prediction--the Phobius web server. *Nucleic acids research* **35**, W429-432.

Kinoshita, S., Udaka, S. & Shimono, M. (2004). Studies on the amino acid fermentation. Part 1. Production of L-glutamic acid by various microorganisms. *The Journal of general and applied microbiology* **50**, 331-343.

Krogh, A., Larsson, B., von Heijne, G. & Sonnhammer, E. L. (2001). Predicting transmembrane protein topology with a hidden Markov model: application to complete genomes. *Journal of molecular biology* **305**, 567-580.

Porollo, A. A., Adamczak, R. & Meller, J. (2004). POLYVIEW: a flexible visualization tool for structural and functional annotations of proteins. *Bioinformatics (Oxford, England)* **20**, 2460-2462.

Snider, C., Jayasinghe, S., Hristova, K. & White, S. H. (2009). MPEx: a tool for exploring membrane proteins. *Protein science : a publication of the Protein Society* **18**, 2624-2628.

Studier, F. W. & Moffatt, B. A. (1986). Use of bacteriophage T7 RNA polymerase to direct selective high-level expression of cloned genes. *Journal of molecular biology* **189**, 113-130.

Tsirigos, K. D., Peters, C., Shu, N., Kall, L. & Elofsson, A. (2015). The TOPCONS web server for consensus prediction of membrane protein topology and signal peptides. *Nucleic acids research* **43**, W401-407.

Tusnady, G. E. & Simon, I. (2001). The HMMTOP transmembrane topology prediction server. *Bioinformatics (Oxford, England)* **17**, 849-850.
